# Dimethylfumarate Suppresses Adipogenic Differentiation in 3T3-L1 Preadipocytes through Inhibition of STAT3 Activity

**DOI:** 10.1371/journal.pone.0061411

**Published:** 2013-04-18

**Authors:** Hyeon-Ji Kang, Hyun-Ae Seo, Younghoon Go, Chang Joo Oh, Nam Ho Jeoung, Keun-Gyu Park, In-Kyu Lee

**Affiliations:** 1 Department of Biomedical Science, Graduate School, Kyungpook National University, Daegu, Republic of Korea; 2 Department of Internal Medicine, Research Institute of Aging and Metabolism, WCU Program, Kyungpook National University School of Medicine, Daegu, Republic of Korea; 3 Departments of Internal Medicine, Daegu Fatima Hospital, Daegu, Republic of Korea; 4 Department of Fundamental Medical & Pharmaceutical Sciences, Catholic University of Daegu, Daegu, Republic of Korea; Georg Speyer Haus, Germany

## Abstract

The excessive accumulation of adipocytes contributes to the development of obesity and obesity-related diseases. The interactions of several transcription factors, such as C/EBPβ, PPARγ, C/EBPα, Nrf2, and STAT3, are required for adipogenic differentiation. Dimethylfumarate (DMF), an immune modulator and antioxidant, may function as an inhibitor of STAT3 and an activator of Nrf2. This study examined whether DMF inhibits adipogenic differentiation of 3T3-L1 preadipocytes by inhibiting STAT3 or activating Nrf2. DMF suppressed 3T3-L1 preadipocyte differentiation to mature adipocytes in a dose-dependent manner as determined by Oil Red O staining. The mRNA and protein levels of adipogenic genes, including C/EBPβ, C/EBPα, PPARγ, SREBP-1c, FAS, and aP2, were significantly lower in DMF-treated 3T3-L1 preadipocytes. Suppression of adipogenic differentiation by DMF treatment resulted primarily from inhibition of the early stages of differentiation. DMF inhibits clonal expansion during adipogenic differentiation through induction of a G1 cell cycle arrest. Additionally, DMF regulates cell cycle-related proteins, such as p21, pRb, and cyclin D. DMF treatment markedly inhibited differentiation medium-induced STAT3 phosphorylation and inhibited STAT3 transcriptional activation of a reporter construct composed of four synthetic STAT3-response elements. Moreover, inhibition of endogenous Nrf2 activity using a dominant negative Nrf2 did not abolish the DMF-induced inhibition of adipogenic differentiation of 3T3-L1 preadipocytes. In summary, DMF is a negative regulator of adipogenic differentiation based on its regulation of adipogenic transcription factors and cell cycle proteins. This negative regulation by DMF is mediated by STAT3 inhibition, but is unlikely to involve Nrf2 activation.

## Introduction

Adipose tissue contributes to the maintenance of energy homeostasis [1] and is considered to be an endocrine organ that contributes to the pathogenesis of obesity and obesity-related metabolic complications [1]. Excessive accumulation of adipose tissue in the body may cause the development of obesity and obesity-related diseases [2]. The accumulation of adipose tissue results from increases both in the size and number of adipocytes [3]. In addition, recent evidence has demonstrated that accelerated adipogenic differentiation is implicated in the excessive accumulation of body fat [4]. Adipogenic differentiation is a complex process accompanied by changes in cytoarchitecture, signaling pathways, and transcriptional regulation. The interactions of several transcription factors, such as peroxisome proliferator-activated receptor gamma (PPARγ), CCAATT enhancer binding proteins (C/EBP), and SREBP-1c, are required for adipogenic differentiation [4], [5].

In addition to these transcription factors, recent studies have shown that the signal transducer and activator of transcription 3 (STAT3) and NF-E2-related factor 2 (Nrf2) play important roles in adipogenic differentiation [6]–[9]. STAT3 is a transcription factor and is required for gp130-mediated cell survival and the G1/S transition in the cell cycle [10]. The transition from G1 to S phase in the cell cycle requires the activation of complexes of cyclin-dependent kinases (CDKs) [11]. In the HepG2 hepatoma cell line, STAT3 regulates the G1/S phase transition through interactions with p21, a potent CDK inhibitor [12]. In 3T3-L1 preadipocytes, STAT3 regulates adipogenesis via regulation of PPARγ and C/EBPβ [6], [7]. Adipogenic differentiation can be suppressed by STAT3 siRNA or a dominant negative STAT3 and the PPARγ agonist rescued adipogenesis in these treatments [6]. Recently, STAT3 was reported to regulate the transcription of C/EBPβ by binding the distal region of the C/EBPβ promoter [7]. By contrast, Nrf2, a basic leucine zipper (bZIP) transcription factor, induces the expression of genes including those related to antioxidant enzymes [13]. Several lines of evidence suggest that Nrf2 activation impairs lipid accumulation in adipose tissue and inhibits adipocyte differentiation [8], [9]. Nrf2 activation diminished during adipogenic differentiation of the bone marrow-derived ST2 cell line [14] and activation of Nrf2 was suggested to inhibit adipogenesis by modulating signaling by the aryl hydrocarbon receptor in experiments using a pharmacological activator of Nrf2 [8]. More recently, enhanced Nrf2 activity was shown to inhibit lipid accumulation in white adipose tissue in leptin-deficient mice [9].

DMF is the active ingredient of an oral formulation of fumaric acid esters with proven effectiveness in patients with chronic plaque psoriasis, a dermatological disorder associated with immune dysfunction [15], [16]. Since the 1950s, DMF has been proven effective in treatment of psoriasis, and several studies have revealed that DMF is also effective in treating multiple sclerosis, inflammatory lung disease, and other conditions [17], [18]. As an immune modulator, DMF decreased synthesis of proinflammatory mediators such as TNF-α, IL-1β, and IL-6 in microglial and astrocytic cells [19]. Because activation of STAT3 is induced by cytokines such as IL-6 and IL-10 [20], [21], DMF may have the potential to function as a STAT3 inhibitor. Moreover, recent reports have shown that DMF increases the expression of Nrf2, which is repressed by binding to the inhibitor Keap1 in the cytoplasm [22]–[24]. Collectively, these data suggest that DMF could modulate adipogenic differentiation.

Here, the potential role of DMF in adipogenic differentiation and the molecular mechanisms by which DMF inhibits adipogenic differentiation, either through inhibiting STAT3 or activating Nrf2, were investigated.

## Results

### DMF Inhibits Adipogenic Differentiation of 3T3-L1 Preadipocytes

**Figure 1:**
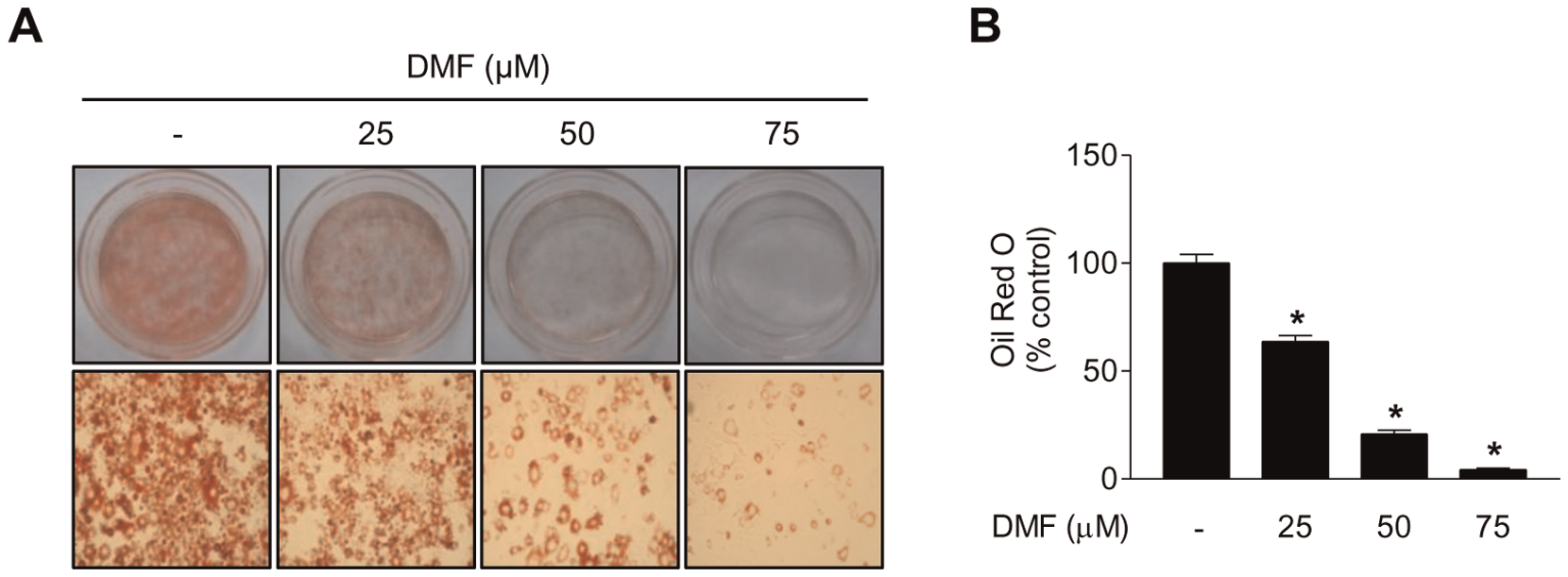
Effect of DMF on adipogenic differentiation. Two-day post-confluent 3T3-L1 preadipocytes were cultured with differentiation medium in the absence or presence of DMF (25–75 µM) for 8 days. (A) Representative micrographs showing cell culture dishes and the cell monolayers stained with Oil Red O (× 200). At day 8, intracellular lipid accumulation was determined by Oil Red O staining. (B) Quantitative analysis of Oil Red O staining. Oil Red O stained cells were extracted with isopropyl alcohol and the absorbance at 500 nm was measured. Data are presented as mean ± S.D. (n = 3); *p<0.001 vs. control.

To determine the effect of DMF on adipogenic differentiation, intracellular lipid accumulation was monitored with an Oil Red O staining assay. Post-confluent 3T3-L1 preadipocytes treated with differentiation medium (MDI), which contains a mixture of IBMX, dexamethasone, and insulin, initiated adipogenic differentiation and Oil Red O staining showed that intracellular lipid accumulation was marked by day 8, suggesting that 3T3-L1 preadipocytes differentiate into mature adipocytes. However, cells co-treated with 25 µM DMF for 8 days showed significant inhibition of MDI-induced lipid droplet accumulation, and 75 µM DMF almost completely blocked lipid droplet accumulation (*p*<0.001) (Fig. 1).

### DMF Inhibits Adipogenic Differentiation Associated Gene Expression

**Figure 2:**
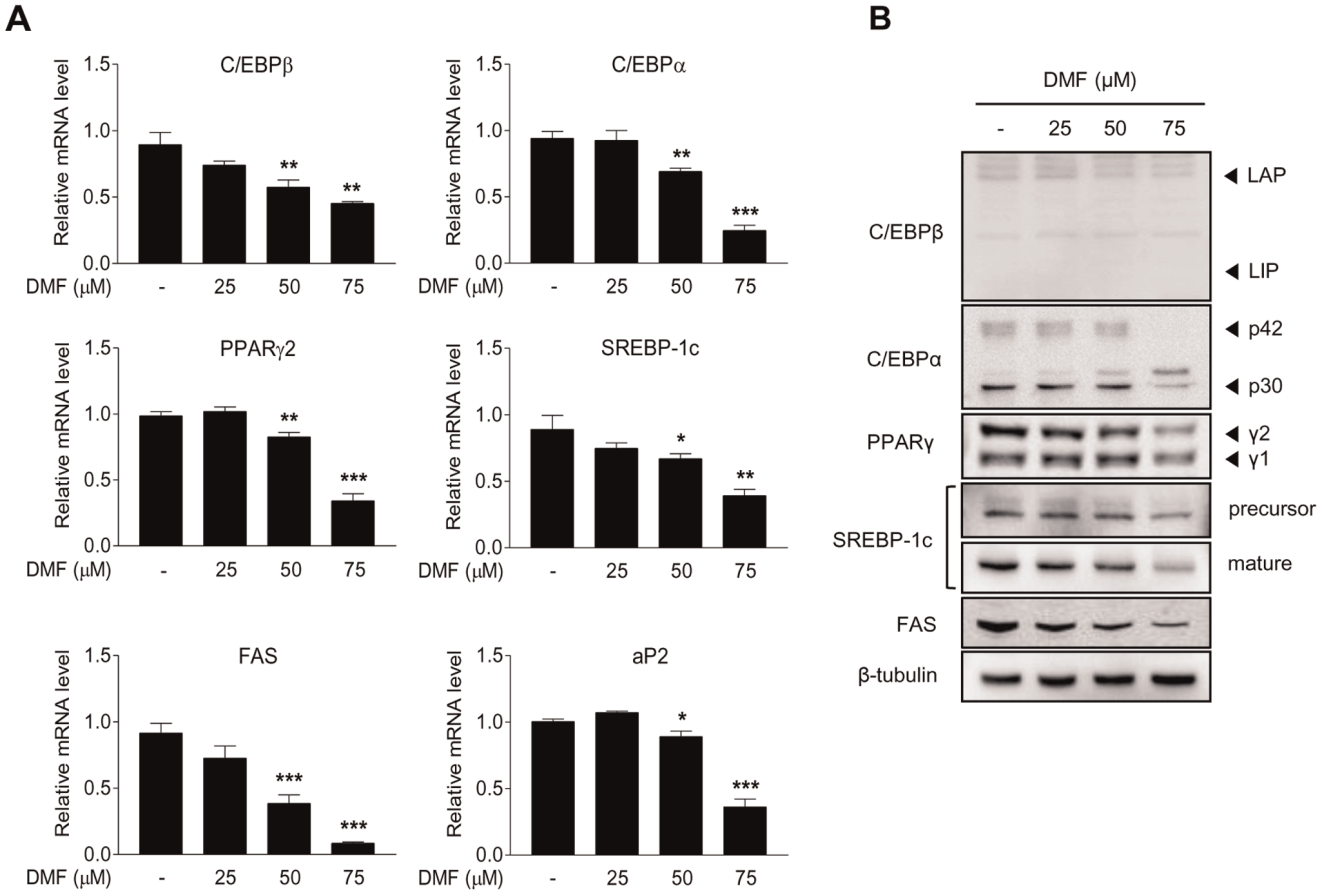
Effect of DMF on adipogenic gene expression in 3T3-L1 cells. Two-day post-confluent 3T3-L1 preadipocytes were subjected to adipogenic differentiation for 8 days in the absence or presence of various concentrations of DMF. (A) Real-time PCR was used to quantify the effect of DMF on adipogenic gene expression. On day 8, adipogenic markers were analyzed by real-time PCR using mRNA isolated from DMF-treated 3T3-L1 cells. (B) Representative Western blot analysis showing the effect of DMF on C/EBPβ, C/EBPα, PPARγ, and SREBP-1c levels. Data are presented as mean ± S.D. (n = 3); *p<0.05, **p<0.01, ***p<0.001 vs. control.

The effect of DMF on the expression levels of genes associated with adipogenic differentiation was investigated. Post-confluent 3T3-L1 preadipocytes were treated with various doses of DMF (0, 25, 50, and 75 µM) during MDI-induced adipogenic differentiation for 8 days. The effects of DMF on adipogenic gene expression were measured by real-time PCR. The mRNA levels of adipogenic genes including C/EBPβ, C/EBPα, PPARγ, SREBP-1c, FAS, and aP2 were significantly lower in DMF-treated 3T3-L1 preadipocytes. The reduction in mRNA levels of these genes was proportional to the DMF concentration (Fig. 2A). The protein levels of the gene products were examined by Western blot analysis. Consistent with the expression patterns, C/EBPβ protein levels were weakly detected and further decreased by DMF treatment. The protein levels of C/EBPα, PPARγ, SREBP-1c, and FAS in DMF-treated 3T3-L1 preadipocytes were decreased by DMF treatment in a dose-dependent manner (Fig. 2B).

### DMF Inhibits Differentiation of 3T3-L1 Preadipocytes during the Early Stages of Adipogenesis

**Figure 3:**
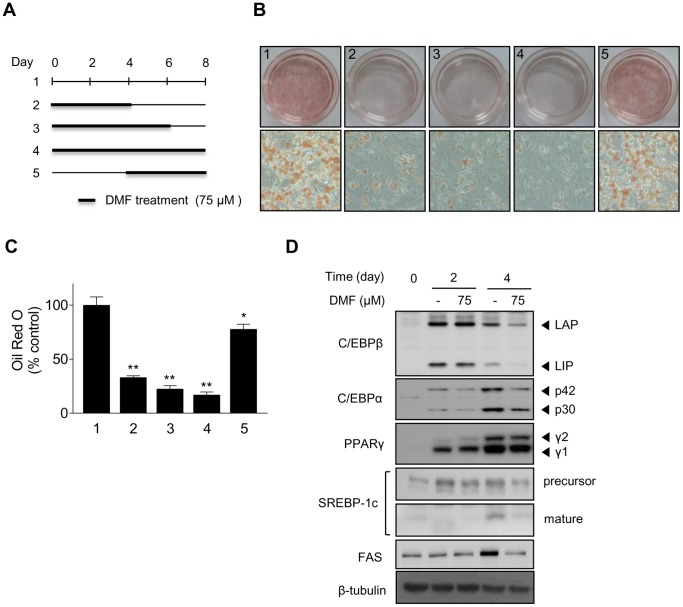
Inhibitory effect of DMF during the early stages of adipogenic differentiation. 3T3-L1 cells were treated with 75 µM DMF for the indicated time periods during 3T3-L1 preadipocyte differentiation. (A) Schematic model depicting DMF treatment during 3T3-L1 preadipocyte differentiation. Thick lines indicate the treatment time at 75 µM DMF. 3T3-L1 cells were treated with 75 µM DMF for the indicated times during 3T3-L1 preadipocyte differentiation. (B) Representative micrographs showing cell culture dishes and cell monolayers stained with Oil Red O (× 200). (C) Quantitative analysis of Oil Red O staining. Oil Red O stained cells were extracted with isopropyl alcohol and the absorbance at 500 nm was measured. (D) Western blot analysis showing the protein levels of C/EBPβ, C/EBPα, PPARγ, SREBP-1c (precursor and mature), and FAS in 3T3-L1 cells after 0, 2, or 4 days of DMF treatment. The data are presented as mean ± S.D. (n = 3); *p<0.05, **p<0.001 vs. control.

To identify the critical stage of adipogenic differentiation affected by DMF treatment, differentiating 3T3-L1 preadipocytes were treated with 75 µM DMF at various time points during adipogenic differentiation, as illustrated in Figure 3A. After 8 days of differentiation, cells were subjected to Oil Red O staining and quantitative analysis of intracellular lipids. Treatment with DMF from day 0 to day 8 (treatment 4) showed the highest inhibition of intracellular lipid droplet accumulation. Treatment with DMF from day 0 to day 4 (treatment 2) or from day 0 to day 6 (treatment 3) showed comparatively low levels of intracellular lipid accumulation. Although the longer DMF treatment tended to decrease intracellular lipid accumulation more extensively, the amounts of inhibition were not statistically different between treatments 2, 3, and 4. Moreover, differentiating 3T3-L1 preadipocytes treated with DMF from days 4 to 8 (treatment 5) resulted in weaker inhibition of adipogenic differentiation compared with treatments 2 and 3 (Fig. 3B and C). These results suggested that DMF acts at an early stage of adipogenic differentiation.

To confirm the action of DMF on an early stage of adipogenic differentiation, the protein levels of adipogenic gene products were examined at day 2 and 4 after MDI treatment. As shown in Figure 3D, C/EBPβ expression on day 4 was still up-regulated, although the level was lower than on day 2 after MDI treatment. DMF decreased C/EBPβ expression on day 4 after MDI treatment but it did not decrease C/EBPβ expression on day 2 after MDI treatment. C/EBPα and PPARγ protein production was detected by day 2 and further increased by day 4 after MDI treatment, but these increases were inhibited by DMF treatment. The levels of mature SREBP-1c protein were minimal at day 2 but were clearly detected by day 4. FAS protein levels were markedly increased at day 4 after MDI treatment, but this increase was inhibited by DMF treatment (Fig. 3D).

### DMF Inhibits 3T3-L1 Preadipocyte Clonal Expansion

**Figure 4:**
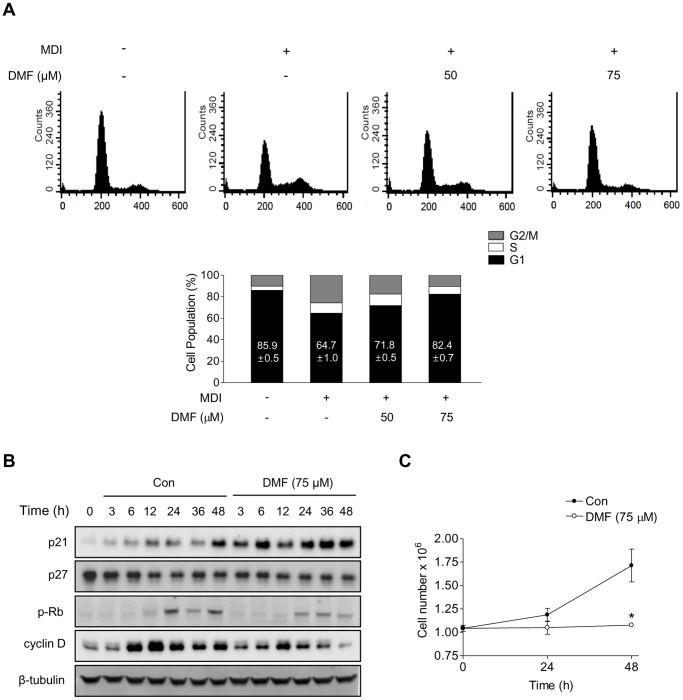
Cell cycle analysis of DMF-treated 3T3-L1 cells during mitotic clonal expansion. (A) Flow cytometric cell cycle analysis. Two-day post-confluent 3T3-L1 preadipocytes were incubated with MDI in the presence or absence of DMF. After 24 h, cells were stained with propidium iodide and subjected to flow cytometric cell cycle analysis (upper panel). The percentage of the cell population at different stages of the cell cycle were calculated from the data in (A) (lower panel). (B) Western blot analysis of cell cycle-related proteins. The time course of p21, p27, pRb, and cyclin D expression was analyzed after incubation in MDI with or without DMF treatment. (C) Relative cell numbers. Cell numbers were determined by hemocytometer counts after incubation with MDI in the presence or absence of DMF. The data are presented as mean ± S.D. (n = 3) *p<0.01.

Mitotic clonal expansion occurs during the early stages of preadipocyte differentiation. Therefore, whether the negative effect of DMF on adipogenic differentiation is the result of inhibition of mitotic clonal expansion was examined. Differentiating 3T3-L1 preadipocytes treated with the indicated concentrations of DMF (0, 50, and 75 µM) were subjected to flow cytometry. As shown in Figure 4A, mitotic clonal expansion in 3T3-L1 preadipocytes was induced by MDI, but DMF treatment effectively induced a G1 cell cycle arrest. The number of cells in the G2/M phase increased in 3T3-L1 preadipocytes in the MDI treatment. However, DMF-treated 3T3-L1 preadipocytes showed a dose-dependent increase in the number of cells in G1 phase and decrease in the number of cells in the G2/M phase (Fig. 4A).

Next, the effect of DMF on the levels of cell cycle-related proteins was examined. The level of p21, a key regulator of cell cycle progression during G1 phase [25], increased in 3T3-L1 preadipocytes treated with DMF for 48 h, compared with cells incubated in the absence of DMF (Fig. 4B). In addition, DMF reduced MDI-induced total cyclin D and phospho-Rb. The levels of p27 were unchanged (Fig. 4B). Consistent with these data, the relative cell numbers were increased by MDI treatment after 1 or 2 days in the medium, but DMF completely prevented the MDI-induced cell proliferation (Fig. 4C). Taken together, these data suggest that DMF inhibits clonal expansion during adipogenic differentiation through the induction of a G1 cell cycle arrest.

### Nrf2 does not Mediate the Anti-adipogenic Differentiation Effect of DMF

**Figure 5:**
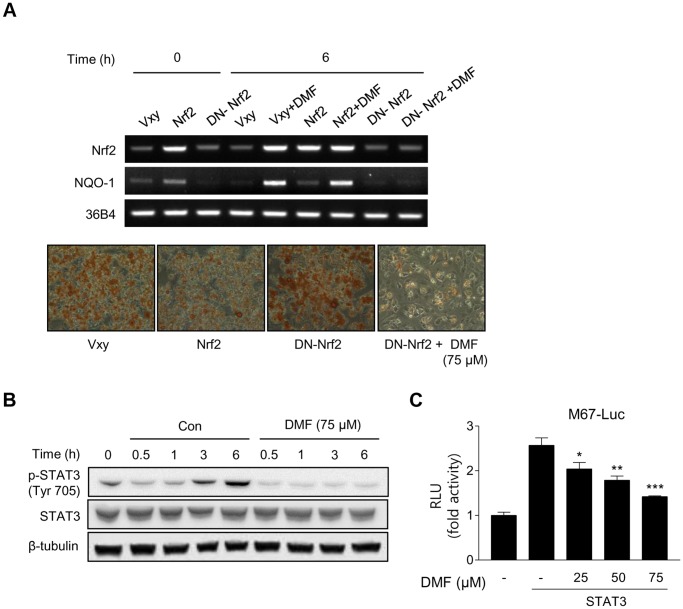
The effect of Nrf2 and STAT3 by DMF on adipogenic differentiation. (A) RT-PCR analysis of 3T3-L1 preadipocytes stably expressing Vxy-puro, Vxy-Nrf2-puro, and Vxy-DN-Nrf2-puro shows the effects of DN-Nrf2 on expression of Nrf2 and its target gene, NQO-1 (upper panel). Oil Red O staining showed the effect of DMF (75 µM) on adipogenesis in Vxy-DN-Nrf2-puro overexpressing 3T3-L1 cells (lower panel). (B) Representative Western blot analysis showing the effect of DMF (75 µM) on STAT3 phosphorylation at Tyr705. Cells were incubated in MDI in the presence or absence of DMF. (C) Transient transfection study with the M67 luciferase reporter. AD-293 cells were transiently transfected with M67-luc and STAT3 expression plasmids. The cells were cultured for 24 h and treated with different concentrations DMF (25–75 µM) for 24 h. Luciferase activity was normalized by β-galactosidase for transfection efficiency. The data are presented as mean ± S.D. (n = 3) *p<0.05, **p<0.01, ***p<0.001 vs. STAT3 transfection.

DMF activates Nrf2, which is known to inhibit adipogenic differentiation [8], [9]. Therefore, whether the effect of DMF on adipogenic differentiation is Nrf2 dependent was examined using cells stably expressing dominant negative (DN) Nrf2. Oil Red O staining indicated that cells stably expressing Nrf2 suppressed adipogenic differentiation and cells stably expressing DN-Nrf2 showed augmented adipogenic differentiation (Fig. 5A). Interestingly, the DMF-induced inhibition of adipogenic differentiation was not abolished in DN-Nrf2 expressing cells, suggesting that DMF inhibition of preadipocyte differentiation is Nrf2 independent. The active function of DN-Nrf2 was confirmed by measuring the expression level of the Nrf2 target gene NQO-1. DMF-induced NQO-1 expression was not found in cells expressing DN-Nrf2 (Fig. 5A).

### STAT3 as a Target for the Anti-adipogenic Differentiation Effect of DMF

A regulatory role for STAT3 during the proliferative phases of adipogenesis has been reported previously [26]. Therefore, whether STAT3 is a target for the anti-adipogenic effect of DMF was examined. As shown in Figure 5B, MDI treatment induced STAT3 phosphorylation but DMF treatment markedly inhibited STAT3 phosphorylation (Fig. 5B). Using a reporter construct composed of four synthetic STAT-response elements (M67-luciferase), the effect of DMF on STAT3 transcriptional activity was examined. Consistent with the Western blot analysis of STAT3 phosphorylation, DMF markedly inhibited STAT3 transcriptional activity (Fig. 5C).

## Discussion

In the present study, DMF was shown to inhibit 3T3-L1 preadipocyte differentiation to adipocytes through inhibition of clonal expansion by the induction of a G1 cell cycle arrest. DMF inhibits transcription factors involved in adipogenic differentiation, including C/EBPβ, C/EBPα, and PPARγ. It appears that DMF inhibits preadipocyte differentiation by down-regulation of STAT3 activity.

Studying adipogenic differentiation using 3T3-L1 preadipocytes is a well-established model system. When 3T3-L1 preadipocytes are incubated in the presence of hormonal inducers, adipogenesis is induced by adipogenesis-related transcription factors such as C/EBPβ, C/EBPα, and PPARγ [27]. C/EBPβ, C/EBPα, and PPARγ are induced by early, before the transcriptional activation of most adipogenic genes, and these factors enhance the expression of genes associated with adipogenic differentiation [28], [29]. This study showed that DMF inhibited 3T3-L1 preadipocyte differentiation and decreased adipogenic gene expression early in differentiation. After treatment of preadipocytes with MDI, a rapid and transient increase in the expression of C/EBPβ is observed. Within 2 days, C/EBPβ reaches its peak level and then begins to decrease, coincident with a rise in C/EBPα and PPARγ expression [30], [31]. C/EBPα is undetectable at the beginning of the differentiation process in preadipocytes, becomes detectable 2 days after MDI stimulation, and reaches its highest levels by 5 days after initiation of the adipogenic differentiation program [32], [33]. PPARγ is induced during the 2 days following induction of adipogenic differentiation and its expression peaks by day 3–4 [34]. Consistent with previous studies, our data showed the protein levels of C/EBPα and PPARγ were weakly detected at day 2, but became abundant by day 4 after MDI stimulation. The C/EBPβ protein level was strongly detected on day 2. The level on day 4 was still markedly up-regulated although it was slightly lower than on day 2. The increase in the C/EBPβ protein level was inhibited by DMF treatment. Collectively, these data suggest that the inhibition of adipogenic differentiation by DMF occurs primarily in the early stages of differentiation.

Preadipocytes differentiate into adipocytes through growth arrest, clonal expansion, early changes in gene expression, later events such as lipid accumulation, and are finally terminally differentiated [4]. In this chronology, mitotic clonal expansion occurs in the early stages of differentiation [4]. Mitotic clonal expansion consists of the two sequential rounds of mitosis which are completed 48–60 h after the induction of adipogenic differentiation [35]. Because the inhibitory effects of DMF occurred mainly within the first 4 days, we investigated the effect of DMF on mitotic clonal expansion of adipocytes. Flow cytometry showed that DMF induced a G1 cell cycle arrest. The present study showed that DMF-treated 3T3-L1 preadipocytes showed elevated levels of p21, which is a potent cyclin-dependent kinase inhibitor and functions as a regulator of the G1 phase of the cell cycle. DMF also inhibited pRb by down-regulation of the cyclin D-dependent kinase, which results in G1 arrest and inhibits clonal expansion.

STAT3 regulates cell growth and differentiation [10] through up-regulation of p21, which induces G1 arrest in the cell cycle [12]. Recent studies demonstrated that STAT3 is associated with adipogenic differentiation. The differentiation of preadipocytes into mature adipocytes was suppressed by inhibition of the STAT3 pathway through the regulation of adipogenic genes such as PPARγ and C/EBPß [6], [7], suggesting that STAT3 may play a role in mediating the effects of DMF on p21 induction and G1 cell cycle arrest. Indeed, DMF inhibited adipogenic MDI-stimulated STAT3 phosphorylation and STAT3 transcriptional activity, as shown using a reporter construct composed of four synthetic STAT-response elements. Because DMF is known to activate Nrf2 [22], the possibility that DMF inhibits adipogenic differentiation via Nrf2 activation was investigated. Inhibition of Nrf2 activity using cells stably expressing DN-Nrf2 increased 3T3-L1 preadipocyte differentiation. However, DN-Nrf2 expressing cells did not attenuate the effect of DMF on adipogenic differentiation, suggesting an Nrf2-independent mechanism.

Recently phase phase III clinical trials of DMF for multiple sclerosis, namely DEFINE and CONFIRM was completed [36], [37]. In these trials, DMF significantly reduced the relapse rates of multiple sclerosis, but changes in body weight were not investigated. In most animal studies of the beneficial effect of DMF, weight changes were not included as part of the data [22], [38]. Therefore, it is possible and necessary to evaluate the effects of DMF on body weight and adipogenesis in human with obesity, especially as DMF was generally well tolerated in previous clinical trials.

In summary, these data demonstrate that DMF is a negative regulator of preadipocyte differentiation through the regulation of adipogenic transcriptional factors and cell cycle proteins. This negative regulation by DMF is mediated by STAT3 inhibition but is unlikely to involve Nrf2 activation.

## Materials and Methods

### Reagents

Dulbecco’s modified Eagle’s medium was purchased from Hyclone (Logan, UT). Dimethylfumarate (DMF), insulin, 3-isobutyl-1-metylxanthine (IBMX), dexamethasone, and Oil Red O dye were purchased from Sigma-Aldrich (St Louis, MO, USA).

### Cell Culture and Differentiation

Murine 3T3-L1 preadipocytes were cultured in high-glucose DMEM (Dulbecco’s modified Eagle’s medium) supplemented with 10% bovine serum (*Gibco*, Auckland, *NZ*), penicillin (100 µg/ml), and streptomycin (100 µg/ml) in a humidified atmosphere of 5% CO_2_ at 37°C. Two days after reaching confluence, 3T3-L1 preadipocytes were induced to differentiate using medium supplemented with 1 µg/ml insulin, 1 µM dexamethasone, 0.5 mM IBMX, and 10% fetal bovine serum (FBS, *Gibco*, US). After 2 days, the medium was replaced with medium containing 10% FBS supplemented with 1 µg/ml insulin, and changed every 2 days thereafter with medium containing 10% FBS.

### Oil Red O Staining

After day 8, differentiated 3T3-L1 preadipocytes were fixed with 10% formalin in PBS for 1 h and washed with 60% isopropyl alcohol. After drying at room temperature, cells were stained with filtered Oil Red O solution for 30 minutes. Stained cells were washed four times with distilled water. The phenotypic changes of adipogenic differentiation were observed using an inverted phase-contrast microscope (*Olympus LX81* **,** Japan). To quantify the amount of Oil Red O stained lipids, stained cells were eluted with 100% isopropyl alcohol for 10min and the absorbance of the extracts was measured at 500 nm in a *VersaMax microplate reader (*Molecular Devices, Sunnyvale, CA, USA).

### Western Blot Analysis

At the indicated times, cells were washed with twice with PBS and lysed with lysis buffer [20 mM Tris (pH 7.4), 5 mM EDTA (pH 8.0), 10 mM Na_4_P_2_OH, 100 mM NaF, 2 mM Na_3_VO_4_, 1% NP-40 and 0.1 mM PMSF] containing proteinase and phosphatase inhibitors. Proteins were separated by SDS-PAGE, transferred to a PVDF membrane (Millipore, USA), and incubated with specific primary antibodies. The antibodies used in this study were directed against SREBP-1c, p21, p27 (BD Biosciences, San Jose, CA, USA), FAS, C/EBPα, PPARγ, Cyclin D, pRb, STAT3, phospho-STAT3 (Cell Signaling, Beverly, MA, USA), C/EBPβ (Santa Cruz Biotechnology, CA, USA). Detection of each protein was performed using ECL (Immobilon™ Western Chemiluminescent HRP Substrate, Millipore) according to the manufacturer’s instructions.

### RT-PCR and Real-time PCR

Total RNA was extracted using the Trizol reagent (Invitrogen, Carlsbad, CA USA) and an aliquot (2 µg) of total RNA was reversed transcribed using the RevertAid^TM^First Strand cDNA Synthesis Kit (Fermentas, Vilnius, Lithuania) according to the manufacturer’s instructions. First strand cDNAs were amplified by PCR using gene-specific primers. Real-time PCR was carried out using SYBR Green (SYBR Green Master Mix, Applied Biosystems, Warrington, UK) with the StepOne™ Real-Time PCR system (Applied Biosystems). The sequences for the primers are shown in Table 1. The expression level of mouse 36B4 was used as the internal control.

**Table 1:** Primer sequences for RT-PCR and Real-time PCR.

Gene	Sequences (5′→ 3′) of the RT-PCR primers
mouse Nrf2	sense - CCATTTACGGAGACCCACCGC
	antisense - GCCCAAGTCTTGCTCCAGCTC
mouse NQO-1	sense - TCGGAGAACTTTCAGTACCC
	antisense - AGGCTAAGCTTGGGAAAAGAAA
mouse 36B4	sense –TGCCACACTCCATCATCAAT
	antisense - CGAAGAGACCGAATCCCATA
**Gene**	**Sequences (5′→ 3′) of the Real-time PCR primers**
mouse C/EBPβ	sense - AGCGGCTGCAGAAGAAGGT
	antisense - GGCAGCTGCCTTGAACAAGTTC
mouse C/EBPα	sense - GCGCAAGAGCCGAGATAAAG
	antisense - CGGTCATTGTCACTGGTCAACT
mouse PPARγ2	sense - CACAAGAGCTGACCCAATGGT
	antisense - GATCGCACTTTGGTATTCTTGGA
mouse SREBP-1c	sense - CCCTACCGGTCTTCTATCAATGA
	antisense - GCAGATTTATTCAGCTTTGCTTCA
mouse FAS	sense - ACCTGGTAGACCACTGCATTGAC
	antisense - CCTGATGAAACGACACATTCTCA
mouse aP2	sense - CCATCCGGTCAGAGAGTACTTTTAA
	antisense - CGAATTCCACGCCCAGTT
mouse 36B4	sense - ACCTCCTTCTTCCAGGCTTT
	antisense - CTCCAGTCTTTATCAGCTGC

### FACS Analysis

To determine the effect of DMF on cell cycle progression, two-day post-confluent preadipocytes were treated with an adipogenic cocktail in the presence or absence of 50 or 75 µM of DMF for 24 h. Cells were fixed with 70% cold ethanol overnight at –20°C and then washed twice with 1×PBS. Cells were analyzed using a FACS Calibur system (BD Bioscience, San Jose, CA, USA) after staining with 1 ml of cold propidiumiodide (PI) solution containing 40 µg/ml propidium iodide, 10 µg/ml of RNase A and 0.1% NP-40.

### Retroviral Plasmids and Retroviral Infection

The pcDNA3-Nrf2 construct was the kind gift of Mi-Kyoung Kwak (Yeungnam University, Korea). The dominant negative mutant of Nrf2 (DN-Nrf2), lacking the N-terminal transcriptional activation domain, was generated by deleting amino acid residues 1–392 [39]. Full-length cDNAs of mouse Nrf2 and DN-Nrf2 were amplified by PCR and inserted into the XhoI and NotI site of the Vxy-puro retroviral vector. The constructs (Vxy-puro, Vxy-Nrf2-puro, and Vxy-DN-Nrf2-puro) were transfected into the Phoenix ecotropic packaging cells using Lipofectamine™ 2000 reagent (Invitrogen). Viral supernatants were collected after 48 h, clarified by filtration through 0.45 µm pore size syringe filters (Sartorius Stedim Biotech, Bohemia, NY, USA), and used for infection of 3T3-L1 preadipocytes in the presence of 5 µg/ml polybrene (Sigma) for 24 h. Infected 3T3-L1 cells were selected with 3 µg/ml puromycin (Sigma) for 7 days.

### Luciferase Reporter Gene Assay

M67-luc and the constructs expressing wild type STAT3 (STAT3-FLAG) were the kind gifts of Dr. James E. Darnell (The Rockefeller University, New York, NY). For luciferase reporter gene assays, AD-293 cells plated in 24-well plates (70% confluence) were co-transfected with the STAT3 expression plasmid and 400 ng/well of M67-luc using the TransIT-LT1 transfection reagent (MirusBio Incorporation, Madison, WI). Cytomegalovirus (CMV)–β-galactosidase (50 ng/well) was co-transfected as an internal control for transfection efficiency. At 24 h post-transfection, the medium was replaced with 0.5% FBS medium with or without DMF and the cells were incubated for 24 h. Cell were lysed for luciferase and β-galactosidase assays and luciferase activity was normalized by the β-galactosidase activity.

### Statistical Analysis

Data are expressed as mean ± S.D. Statistical analyses were performed using an unpaired Student’s t-test and a value of *P*<0.05 was considered to be significant.
